# 3,9-Dibromo-6,7-dihydro-5*H*-dibenzo[*c*,*e*]thiepine

**DOI:** 10.1107/S1600536808013226

**Published:** 2008-05-10

**Authors:** Hai-Quan Zhang, Bao-Li, Guang-Di Yang, Yu-Guang Ma

**Affiliations:** a State Key Laboratory of Metastable Materials Science and Technology, Yanshan University, Qinhuangdao 066004, People’s Republic of China; b State Key Laboratory of Supramolecular Structures and Materials, Jilin University, Changchun 130012, People’s Republic of China

## Abstract

In the title mol­ecule, C_14_H_10_Br_2_S, the two benzene rings form a dihedral angle of 48.35 (14)°. The seven-membered ring adopts a boat conformation. In the crystal structure, mol­ecules are related by translation along the *b* axis and exhibit C—H⋯π inter­actions.

## Related literature

For the synthesis of dibenzo[*c*,*e*]thiepine derivatives, see: Truce *et al.* (1956 ▶). For the chiroptical properties of dibenzo[*c*,*e*]thiepine derivatives, see: Tomascovic *et al.* (2000 ▶), respectively.
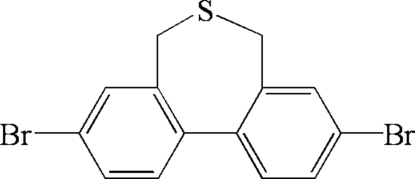


## Experimental

### 

#### Crystal data

C_14_H_10_Br_2_S
*M* *_r_* = 370.10
Monoclinic, 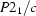

*a* = 8.6629 (12) Å
*b* = 4.7219 (5) Å
*c* = 30.867 (3) Å
β = 93.720 (5)°
*V* = 1260.0 (3) Å^3^
*Z* = 4
Mo *K*α radiation
μ = 6.57 mm^−1^
*T* = 291 (2) K
0.16 × 0.14 × 0.13 mm

#### Data collection

Rigaku R-AXIS RAPID diffractometer
Absorption correction: multi-scan (*ABSCOR*; Higashi, 1995 ▶) *T*_min_ = 0.419, *T*_max_ = 0.482 (expected range = 0.370–0.426)
4858 measured reflections
2850 independent reflections
1840 reflections with *I* > 2σ(*I*)
*R*_int_ = 0.045

#### Refinement

*R*[*F* ^2^ > 2σ(*F* ^2^)] = 0.038
*wR*(*F* ^2^) = 0.078
*S* = 1.01
2850 reflections
154 parameters
H-atom parameters constrained
Δρ_max_ = 0.79 e Å^−3^
Δρ_min_ = −0.54 e Å^−3^

### 

Data collection: *RAPID-AUTO* (Rigaku, 1998 ▶); cell refinement: *RAPID-AUTO*; data reduction: *CrystalStructure* (Rigaku/MSC and Rigaku, 2002 ▶); program(s) used to solve structure: *SHELXS97* (Sheldrick, 2008 ▶); program(s) used to refine structure: *SHELXL97* (Sheldrick, 2008 ▶); molecular graphics: *PLATON* (Spek, 2003 ▶); software used to prepare material for publication: *SHELXL97*.

## Supplementary Material

Crystal structure: contains datablocks global, I. DOI: 10.1107/S1600536808013226/cv2397sup1.cif

Structure factors: contains datablocks I. DOI: 10.1107/S1600536808013226/cv2397Isup2.hkl

Additional supplementary materials:  crystallographic information; 3D view; checkCIF report

## Figures and Tables

**Table 1:** Hydrogen-bond geometry (Å, °)

*D*—H⋯*A*	*D*—H	H⋯*A*	*D*⋯*A*	*D*—H⋯*A*
C14—H14a⋯*Cg*^i^	0.97	2.69	3.446 (9)	136

Symmetry code: (i) 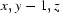. *Cg* is the centroid of the benzene ring.

**Fig. 1.**
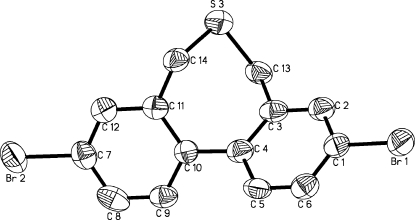
The molecular structure of (I) showing the atomic numbering and 40% probability displacement ellipsoids.

## supplementary crystallographic information

### Comment

The dibenzo[c,e]thiepine derivatives (Truce *et al.* 1956) exhibit remarkable chiroptical properties (Tomascovic *et al.* 2000). Introducing Br on benzene ring of dibenzo[c,e]thiepine can expand the field of their application, such as photoluminescence, electro-luminescence devices and nonlinear potics *etc*. Herein we present the crysal structure of the title compound, (I).

The molecular structure of (I) is shown in Fig. 1. The molecule exhibits twisted conformation with a dihedral angle of 48.35 (14)° between two benzene rings, while central 7-member ring has a boat conformation. The crystal structure of (I) is stabilized by C—H···π interactions (Table 1).

### Experimental

The title compound has been prepared in a four-step reaction.   Step 1: the 2,7-dibromophenanthrenequinone was obtained by direct bromination of phenanthrenequinone in the presence of n-bromosuccinimide in H_2_SO_4_. Step 2: the oxidation of 2,7-dibromophenanthrenequinone in the presence of pure oxygen and Cu(I)Cl gave 4,4-dibromodiphenic acid. Step 3: the reduction of 4,4-dibromodiphenic acid using NaBH_4_ gave 4,4'-dibromo-2,2'-bis-(hydroxymethyl)-bipheny. Step 4: the reacton of 4,4'-dibromo-2,2'-bis-(hydroxymethyl)-biphenyl and sodium sulfate nonahydrate gave the title compound. Crystals suitable for single-crystal X-ray diffraction were grown by slow evaporation of a ethanol solution.

### Refinement

H atoms were geometrically positioned (C—H = 0.93–0.97 Å) and refined as riding, with *U*_iso_(H) = 1.2–1.5*U*_eq_(C).

### Crystal data

**Table:**

C_14_H_10_Br_2_S	*F*_000_ = 720
*M**_r_* = 370.10	*D*_x_ = 1.951 Mg m^−^^3^
Monoclinic, *P*2_1_/*c*	Mo *K*α radiation λ = 0.71073 Å
Hall symbol: -P 2ybc	Cell parameters from 3351 reflections
*a* = 8.6629 (12) Å	θ = 5.0–54.9º
*b* = 4.7219 (5) Å	µ = 6.57 mm^−^^1^
*c* = 30.867 (3) Å	*T* = 291 (2) K
β = 93.720 (5)º	Block, colourless
*V* = 1260.0 (3) Å^3^	0.16 × 0.14 × 0.13 mm
*Z* = 4	

### Data collection

**Table:**

Rigaku R-AXIS RAPID diffractometer	2850 independent reflections
Radiation source: fine-focus sealed tube	1840 reflections with *I* > 2σ(*I*)
Monochromator: graphite	*R*_int_ = 0.045
*T* = 291(2) K	θ_max_ = 27.5º
ω scans	θ_min_ = 2.6º
Absorption correction: multi-scan(ABSCOR; Higashi, 1995)	*h* = −11→11
*T*_min_ = 0.420, *T*_max_ = 0.482	*k* = −6→6
4858 measured reflections	*l* = −40→40

### Refinement

**Table:**

Refinement on *F*^2^	Secondary atom site location: difference Fourier map
Least-squares matrix: full	Hydrogen site location: inferred from neighbouring sites
*R*[*F*^2^ > 2σ(*F*^2^)] = 0.038	H-atom parameters constrained
*wR*(*F*^2^) = 0.078	*w* = 1/[σ^2^(*F*_o_^2^) + (0.0319*P*)^2^] where *P* = (*F*_o_^2^ + 2*F*_c_^2^)/3
*S* = 1.01	(Δ/σ)_max_ < 0.001
2850 reflections	Δρ_max_ = 0.79 e Å^−^^3^
154 parameters	Δρ_min_ = −0.54 e Å^−^^3^
Primary atom site location: structure-invariant direct methods	Extinction correction: none

### Special details

**Table:**

Geometry. All e.s.d.'s (except the e.s.d. in the dihedral angle between two l.s. planes) are estimated using the full covariance matrix. The cell e.s.d.'s are taken into account individually in the estimation of e.s.d.'s in distances, angles and torsion angles; correlations between e.s.d.'s in cell parameters are only used when they are defined by crystal symmetry. An approximate (isotropic) treatment of cell e.s.d.'s is used for estimating e.s.d.'s involving l.s. planes.
Refinement. Refinement of *F*^2^ against ALL reflections. The weighted *R*-factor *wR* and goodness of fit *S* are based on *F*^2^, conventional *R*-factors *R* are based on *F*, with *F* set to zero for negative *F*^2^. The threshold expression of *F*^2^ > σ(*F*^2^) is used only for calculating *R*-factors(gt) *etc*. and is not relevant to the choice of reflections for refinement. *R*-factors based on *F*^2^ are statistically about twice as large as those based on *F*, and *R*- factors based on ALL data will be even larger.

### Fractional atomic coordinates and isotropic or equivalent isotropic displacement parameters (Å^2^)

**Table:**

	*x*	*y*	*z*	*U*_iso_*/*U*_eq_	
Br1	0.41895 (5)	0.61945 (10)	0.055440 (15)	0.03375 (14)	
Br2	1.35085 (6)	1.76323 (11)	0.226693 (14)	0.03785 (14)	
S3	1.09057 (13)	1.1401 (3)	0.04915 (4)	0.0327 (3)	
C12	1.2114 (5)	1.4013 (9)	0.16192 (12)	0.0249 (9)	
H12A	1.3131	1.3583	0.1564	0.030*	
C1	0.5734 (5)	0.8581 (9)	0.08152 (14)	0.0275 (10)	
C5	0.6896 (5)	1.0665 (9)	0.14581 (13)	0.0276 (10)	
H5A	0.6905	1.0957	0.1756	0.033*	
C11	1.0890 (4)	1.2718 (9)	0.13757 (12)	0.0229 (9)	
C9	0.9122 (5)	1.5295 (9)	0.17965 (12)	0.0264 (10)	
H9A	0.8115	1.5724	0.1861	0.032*	
C2	0.6805 (4)	0.9909 (9)	0.05755 (13)	0.0267 (9)	
H2A	0.6759	0.9670	0.0276	0.032*	
C3	0.7955 (5)	1.1598 (9)	0.07744 (13)	0.0258 (10)	
C7	1.1815 (5)	1.5920 (9)	0.19398 (13)	0.0278 (10)	
C10	0.9356 (4)	1.3383 (9)	0.14641 (12)	0.0217 (9)	
C8	1.0326 (5)	1.6569 (9)	0.20317 (13)	0.0318 (11)	
H8A	1.0137	1.7854	0.2250	0.038*	
C6	0.5753 (5)	0.9018 (9)	0.12604 (13)	0.0284 (10)	
H6A	0.5000	0.8204	0.1422	0.034*	
C14	1.1210 (5)	1.0451 (9)	0.10551 (12)	0.0270 (10)	
H14A	1.0559	0.8835	0.1110	0.032*	
H14B	1.2277	0.9850	0.1108	0.032*	
C4	0.8037 (5)	1.1905 (9)	0.12287 (13)	0.0267 (10)	
C13	0.9066 (4)	1.3159 (9)	0.05066 (12)	0.0264 (10)	
H13A	0.9229	1.5046	0.0625	0.032*	
H13B	0.8611	1.3355	0.0213	0.032*	

### Atomic displacement parameters (Å^2^)

**Table:**

	*U*^11^	*U*^22^	*U*^33^	*U*^12^	*U*^13^	*U*^23^
Br1	0.0284 (2)	0.0346 (3)	0.0382 (3)	0.0011 (2)	0.00183 (18)	−0.0060 (2)
Br2	0.0429 (3)	0.0450 (3)	0.0250 (2)	−0.0075 (2)	−0.00297 (19)	−0.0022 (2)
S3	0.0311 (6)	0.0410 (7)	0.0266 (5)	0.0019 (5)	0.0057 (5)	−0.0010 (5)
C12	0.027 (2)	0.023 (2)	0.024 (2)	0.001 (2)	0.0027 (17)	0.006 (2)
C1	0.025 (2)	0.025 (2)	0.032 (2)	0.009 (2)	0.0002 (18)	−0.004 (2)
C5	0.030 (2)	0.033 (3)	0.021 (2)	0.005 (2)	0.0075 (17)	−0.0017 (19)
C11	0.027 (2)	0.021 (2)	0.0209 (19)	0.0035 (19)	0.0031 (17)	0.0046 (19)
C9	0.029 (2)	0.026 (2)	0.025 (2)	0.0064 (19)	0.0061 (18)	0.0033 (19)
C2	0.026 (2)	0.030 (2)	0.024 (2)	0.006 (2)	0.0037 (18)	0.002 (2)
C3	0.028 (2)	0.024 (2)	0.026 (2)	0.0075 (19)	0.0071 (18)	0.0032 (19)
C7	0.033 (2)	0.028 (2)	0.022 (2)	−0.004 (2)	−0.0004 (18)	0.005 (2)
C10	0.023 (2)	0.022 (2)	0.020 (2)	0.0029 (18)	0.0016 (16)	0.0039 (17)
C8	0.048 (3)	0.025 (3)	0.023 (2)	0.000 (2)	0.009 (2)	0.0008 (19)
C6	0.027 (2)	0.030 (2)	0.029 (2)	−0.001 (2)	0.0065 (18)	0.005 (2)
C14	0.024 (2)	0.025 (3)	0.032 (2)	0.0041 (18)	0.0034 (18)	−0.0042 (19)
C4	0.026 (2)	0.029 (3)	0.025 (2)	0.0058 (19)	0.0044 (17)	0.0021 (19)
C13	0.031 (2)	0.030 (3)	0.0186 (19)	0.0030 (19)	0.0047 (17)	0.0019 (18)

### Geometric parameters (Å, °)

**Table:**

Br1—C1	1.889 (4)	C9—C10	1.392 (5)
Br2—C7	1.906 (4)	C9—H9A	0.9300
S3—C14	1.800 (4)	C2—C3	1.388 (6)
S3—C13	1.800 (4)	C2—H2A	0.9300
C12—C7	1.375 (6)	C3—C4	1.407 (5)
C12—C11	1.400 (5)	C3—C13	1.502 (5)
C12—H12A	0.9300	C7—C8	1.373 (6)
C1—C2	1.375 (6)	C10—C4	1.488 (6)
C1—C6	1.389 (6)	C8—H8A	0.9300
C5—C6	1.371 (6)	C6—H6A	0.9300
C5—C4	1.382 (6)	C14—H14A	0.9700
C5—H5A	0.9300	C14—H14B	0.9700
C11—C10	1.409 (5)	C13—H13A	0.9700
C11—C14	1.496 (6)	C13—H13B	0.9700
C9—C8	1.370 (6)		
			
C14—S3—C13	99.46 (18)	C9—C10—C11	118.1 (4)
C7—C12—C11	120.1 (4)	C9—C10—C4	121.4 (4)
C7—C12—H12A	120.0	C11—C10—C4	120.4 (4)
C11—C12—H12A	120.0	C9—C8—C7	119.0 (4)
C2—C1—C6	120.0 (4)	C9—C8—H8A	120.5
C2—C1—Br1	121.8 (3)	C7—C8—H8A	120.5
C6—C1—Br1	118.2 (3)	C5—C6—C1	119.1 (4)
C6—C5—C4	122.2 (4)	C5—C6—H6A	120.4
C6—C5—H5A	118.9	C1—C6—H6A	120.4
C4—C5—H5A	118.9	C11—C14—S3	116.1 (3)
C12—C11—C10	119.3 (4)	C11—C14—H14A	108.3
C12—C11—C14	120.1 (4)	S3—C14—H14A	108.3
C10—C11—C14	120.3 (4)	C11—C14—H14B	108.3
C8—C9—C10	122.2 (4)	S3—C14—H14B	108.3
C8—C9—H9A	118.9	H14A—C14—H14B	107.4
C10—C9—H9A	118.9	C5—C4—C3	118.4 (4)
C1—C2—C3	120.9 (4)	C5—C4—C10	120.0 (4)
C1—C2—H2A	119.5	C3—C4—C10	121.5 (4)
C3—C2—H2A	119.5	C3—C13—S3	112.8 (3)
C2—C3—C4	119.2 (4)	C3—C13—H13A	109.0
C2—C3—C13	120.4 (4)	S3—C13—H13A	109.0
C4—C3—C13	120.4 (4)	C3—C13—H13B	109.0
C8—C7—C12	121.2 (4)	S3—C13—H13B	109.0
C8—C7—Br2	119.8 (3)	H13A—C13—H13B	107.8
C12—C7—Br2	119.0 (3)		

### Hydrogen-bond geometry (Å, °)

**Table:**

*D*—H···*A*	*D*—H	H···*A*	*D*···*A*	*D*—H···*A*
C14—H14a···Cg^i^	0.97	2.69	3.446 (9)	136

Symmetry codes: (i) *x*, *y*−1, *z*.

## References

[bb1] Higashi, T. (1995). *ABSCOR* Rigaku Corporation, Tokyo, Japan.

[bb2] Rigaku (1998). *RAPID-AUTO* Rigaku Corporation, Tokyo, Japan.

[bb3] Rigaku/MSC and Rigaku (2002). *CrystalStructure* MSC, The Woodlands, Texas, USA, and Rigaku Corporation, Tokyo, Japan.

[bb4] Sheldrick, G. M. (2008). *Acta Cryst.* A**64**, 112–122. 10.1107/S0108767307043930 18156677

[bb5] Spek, A. L. (2003). *J. Appl. Cryst.* **36**, 7–13.

[bb6] Tomascovic, L. L., Arneri, R. S., Brundic, A. H., Nagl, A., Mintas, M. & Sandtrom, J. (2000). *Helv. Chim. Acta*, **83**, 479–493.

[bb7] Truce, W. E. & Emrick, D. D. (1956). *J. Am. Chem. Soc.* **78**, 6130–6137.

